# Oxytocin modulates the temporal dynamics of resting EEG networks

**DOI:** 10.1038/s41598-019-49636-6

**Published:** 2019-09-26

**Authors:** Bastian Schiller, Thomas Koenig, Markus Heinrichs

**Affiliations:** 1 Department of Psychology, Laboratory for Biological and Personality Psychology, University of Freiburg, DE-79104 Freiburg, Germany; 2 Freiburg Brain Imaging Center, University Medical Center, University of Freiburg, DE-79104 Freiburg, Germany; 3 Translational Research Center, University Hospital of Psychiatry, University of Bern, CH-3000 Bern, Switzerland

**Keywords:** Social neuroscience, Human behaviour

## Abstract

Oxytocin is a key modulator of social interaction, but we possess little knowledge of its underlying effects on neuropsychological processes. We used a spatio-temporal EEG microstates analysis to reveal oxytocin’s effects on the temporal dynamics of intrinsically generated activity in neural networks. Given oxytocin’s known anxiolytic effects, we hypothesized that it increases the temporal stability of the four archetypal EEG resting networks. Eighty-six male participants had received oxytocin or placebo intranasally before we recorded their resting EEG. As hypothesized, oxytocin globally increased the average duration of the four archetypal resting networks and specifically decreased the occurrence and coverage of an autonomic processing-related network to benefit greater coverage of an attention-related network. Moreover, these neurophysiological changes were more pronounced in participants with high anxiety levels and strong subjectively experienced effects of the oxytocin administration. In sum, our study shows that oxytocin reduces rapid switching among neural resting networks by increasing their temporal stability. Specifically, it seems to reduce the brain’s need for preparing the internally-oriented processing of autonomic information, thus enabling the externally-oriented processing of social information. Changes in the temporal dynamics of resting networks might underlie oxytocin’s anxiolytic effects - potentially informing innovative psychobiological treatment strategies.

## Introduction

In the past 15 years, a plethora of intranasal application studies has identified the hypothalamic neuropeptide oxytocin as a key modulator of social cognition and behavior in humans^1–4^. Yet we still possess little empirical evidence of changes in specific neuropsychological processes underlying oxytocin’s effects, evidence sorely missed in tailoring, monitoring, and evaluating individual treatment applications of this neuropeptide in order to improve social malfunctioning^1,5^. To generate such evidence, we here study oxytocin’s effects on the temporal dynamics of neural networks at rest by applying a spatio-temporal analysis of multichannel electroencephalography^6–8^. The applied analysis approach adds to previous research that has mostly relied on metabolic neuroimaging^2,9–11^ in three respects. First, it examines changes in scalp electrical potential topographies, which indicate changes in global network activity, thereby taking into account that even rather circumscribed neuropsychological processes involve massive parallel processing in distributed neural networks^12^. Second, it examines these networks’ intrinsically generated activity, which optimally prepares the individual for forthcoming stimuli processing^13^, thus enabling us to identify oxytocin’s general neuropsychological effects independent from specific social contexts^14^. Third, it examines the temporal dynamics of neural resting networks which mediate specific neuropsychological processes by undergoing rapid reorganization into different spatial patterns^15^, uniquely revealing oxytocin’s effects on switching among neural resting networks on a milliseconds scale.

Research applying metabolic neuroimaging to reveal oxytocin’s effects on neural processing at rest in the spatial domain has demonstrated modulatory effects on the connectivity between distinct neural regions (e.g., amygdala-prefrontal connectivity^16–18^; cortico-striatal connectivity^19^; striatal/pallidal-frontal and striatal-cerebellar connectivity^20^). Recent studies have also reported oxytocin-induced changes in large-scale neural resting networks, suggesting a modulation of neural communication between attention-, saliency-, and default mode-related networks^11,21^. However, as intrinsically generated brain activity at rest is thought to optimally prepare the individual for forthcoming, often unpredictable and rapidly changing stimulus processing, the reorganizing of neural networks must occur on a sub-second time scale^7^. Therefore, it could be highly informative to supplement knowledge gained from metabolic neuroimaging research with knowledge on oxytocin’s effects on neural processing at rest in the temporal domain by using neuroimaging methods with high temporal resolution, such as electroencephalography (EEG; for a recent example, see^22^).

More specifically, the applied spatio-temporal analysis approach clusters the resting EEG signal into a limited number of scalp electrical potential topographies that remain stable for certain time periods (60–120 ms) before dynamically changing into a different topography that remains stable again (for recent studies taking this approach, see^23,24^). These time periods of stable topographies are known as “microstates”, and transitions between microstates are believed to represent sequential coordinated activity of various, distributed neural networks^6,7^. Remarkably, just four archetypal topographies (termed microstate A-D^25^) explain large portions of the global variance in the EEG data (>70%). These four states have been proven to be highly reproducible across multiple independent studies and may result from evolutionarily determined, brain-intrinsic biases toward particular patterns of co-activation particularly suited to representing environmentally relevant information^7^. The temporal dynamics of these four microstates (average duration = temporal stability, average occurrence = usage, coverage = presence, transitions = communication between networks) are known to vary across various behaviors, personality types, and neuropsychiatric disorders^26^. To interpret these variations, researchers have attempted to link these four microstates to specific underlying neural networks, with microstates A and B being associated with sensory processing (microstate A: audition; microstate B: vision), microstate C with autonomic processing, and microstate D with attention-related processing^7,26^. Analyzing how oxytocin modulates the temporal dynamics of these four archetypal resting networks thus holds the potential to deepen our understanding of its effects on specific neuropsychological processes.

Based on the vast literature on oxytocin research in humans, which hypotheses can one derive regarding its effects on specific neuropsychological processes? Pioneering evidence that oxytocin reduces subjective and neuroendocrine responses to psychosocial stress^27^ has inspired a plethora of studies repeatedly demonstrating oxytocin’s anxiolytic effects^1,2^. Metabolic neuroimaging studies have revealed that these effects are due to changes in the activity in and connectivity between neural regions shown to mediate negative affective experiences (e.g., the amygdala, insula^2,9,10^). Whereas these studies have identified neural target regions of oxytocin’s anxiolytic effects, the fluctuations observed using metabolic neuroimaging occur too slowly to be associated with neural networks’ intrinsically generated activity associated with preparing for a quick and adaptive reaction to potentially threatening stimuli^28,29^. Therefore, the present study utilizes high temporal resolution 64-channel EEG in order to reveal oxytocin-induced modulations of the temporal dynamics of neural resting networks on the milliseconds scale which might mediate oxytocin’s anxiolytic effects. For that purpose, we analyzed eighty-six male participants’ resting EEG, which we recorded 45 minutes after they had received oxytocin or placebo treatment intranasally^27,30,31^.

Given oxytocin’s known anxiolytic effects^1,2^, we hypothesized that it increases neural networks’ temporal stability and induces a shift from internally- to externally-oriented processing modes at rest. Because of the association of microstate C with interoceptive-autonomic processing^32,33^ and the association of hyperactive autonomic processing at rest with anxiety^34,35^, we hypothesized that oxytocin decreases the usage and presence of autonomic processing-related microstate C in favor of attention-related microstate D. We did not expect changes with regard to the usage and presence of sensory-processing-related microstates A and B. On an exploratory level, we also analyzed oxytocin’s effects on the communication (i.e., transitions) between microstates. Furthermore, if these hypothetical changes in neural networks’ temporal dynamics do underlie oxytocin’s anxiolytic effects, we should also observe those changes being moderated by anxiety-related traits (e.g., neuroticism^36^, anxious attachment style^37^, dependent attachment style^37^). Participants with high anxiety levels should benefit more from oxytocin’s anxiolytic effects than those with low anxiety levels (for a review on individual moderators of oxytocin’s effects, see^14^). Finally, we analyzed whether the aforementioned changes in neural networks’ temporal dynamics after oxytocin administration are moderated by subjective ratings of experienced substance effects. Such an association would provide innovative evidence that neurophysiological changes could inform the designing of individualized, therapeutic applications of the neuropeptide.

## Results

### The four archetypal microstates

**Figure 1:**
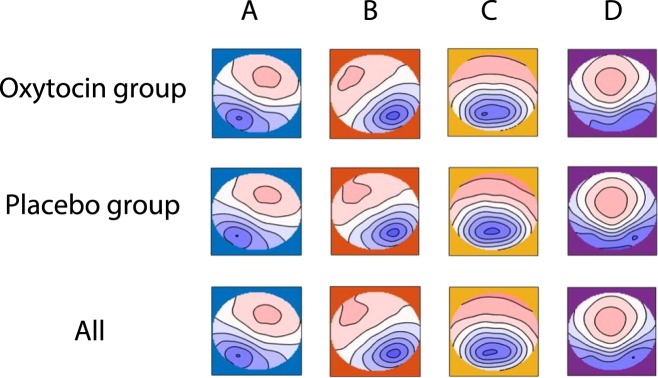
Microstate maps. Topographies of the four archetypal microstates A-D^7,25^, identified in the oxytocin group (first row), the placebo group (second row), and the whole sample (third row). Head seen from above, nose up, left ear left. Red and blue are the arbitrarily chosen color labels for areas of opposite polarity. Please note that the topographies of the four microstates did not differ between treatment groups (all Ps > 0.083).

The applied cluster analysis identified the four archetypal microstate topographies A-D that were exhibited by participants in both the placebo and oxytocin group (Fig. 1). These maps did not differ with respect to their topography between treatment groups (microstate A: *P* > 0.20, microstate B: *P* = 0.157, microstate C: *P* > 0.20; microstate D: *P* = 0.083), which allows us to compare characteristics of the same underlying neural networks between participants in the placebo and oxytocin groups. On average, the four maps explained 78.32% of the variance in our data (s.d. = 3.53%; range: 68–85%).

### Temporal dynamics of resting networks under oxytocin vs. placebo treatment

To compare the temporal dynamics of resting networks (*duration*, *occurrence*, *coverage*, *transitions*) between treatment groups (for descriptive statics see Supplementary Table S1), we calculated ANOVAs with the between-participants factor “treatment” (oxytocin vs. placebo) and the within-participants factor “microstate class” (four levels: microstate A-D) or “microstate class transition” (twelve levels: from microstate A-D [4] to microstate A-D [3]). As the present study focuses on oxytocin’s effects on neural resting networks, we will only report those effects including the “treatment” factor in the following (please see Supplementary Results for main effects of “microstate class” and post-hoc LSD tests).

**Figure 2:**
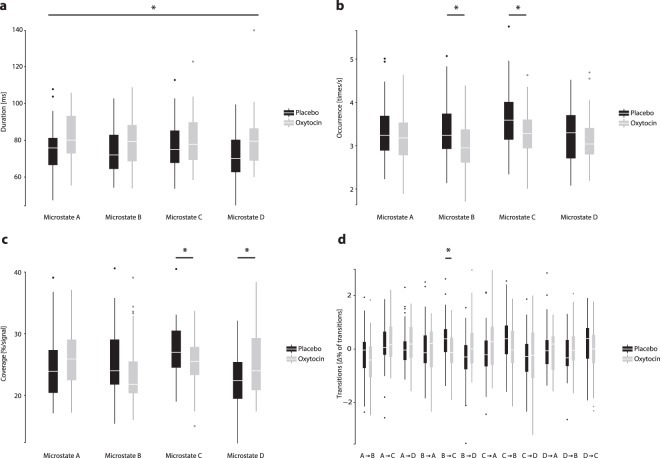
Box-plots (lower end of the whisker: 2.5% percentile; lower horizontal end of the box = 25% percentile; middle horizontal line = median; upper horizontal end of the box = 75% percentile; upper end of the whisker: 97.5% percentile) depicting differences in the temporal dynamics of microstates, i.e., resting networks under oxytocin (grey color) and placebo (black color) treatment. Asterisks indicate significant differences between treatment groups (*P* < 0.10) (**a**) Differences in mean duration in milliseconds (ms). The mean duration of microstates was longer under oxytocin treatment (main effect “treatment”, *P* = 0.015). (**b**) Differences in mean occurrence in times per second. Microstate B (*P* = 0.003) and microstate C (*P* = 0.004) occurred less often under oxytocin treatment (interaction effect of “microstate class x treatment”, *P* = 0.009). (**c**) Differences in coverage in % of signal. Microstate C covered, on a marginally significant level, less time (*P* = 0.054), whereas microstate D covered more time under oxytocin treatment (*P* = 0.027; interaction effect of “microstate class x treatment”, *P* = 0.024). (**d**) Difference in transitions in Δ% (observed-expected; see Methods). There were fewer transitions from microstate B to C under oxytocin treatment (microstate B→C: *P* = 0.004, Bonferroni-corrected; interaction effect of “microstate class transition x treatment”, *P* = 0.050).

Regarding the *duration* of microstates, we observed a significant effect of “treatment” (*F*(1,84) = 6.163, *P* = 0.015, *ETA*^2^ = 0.068), but no interaction effect of “treatment x microstate class” (*F*(3,252) = 1.280, *P* > 0.20). The mean duration of microstates was significantly longer under oxytocin treatment (*M* = 81.1 ms, s.d. = 11.45 ms) than placebo treatment (*M* = 75.2 ms, s.d. = 11.37 ms; Fig. 2a). Thus, as hypothesized, oxytocin increased the temporal stability of resting networks.

Regarding the *occurrence* of microstates, we noted a significant effect of “treatment” (*F*(1,84) = 5.919, *P* = 0.017, *ETA*^2^ = 0.066), and an interaction effect of “microstate class x treatment” (*F*(3,252) = 3.486, *P* = 0.016, *ETA*^2^ = 0.040). Microstate B (*F*(1,84) = 9.379, *P* = 0.003, *ETA*^2^ = 0.100) and microstate C (*F*(1,84) = 8.982, *P* = 0.004, *ETA*^2^ = 0.097) occurred less often under oxytocin treatment (microstate B: *M* = 2.99/s, s.d. = 0.56/s; microstate C: *M* = 3.21/s, s.d. = 0.58/s) than placebo treatment (microstate B: *M* = 3.40/s, s.d. = 0.66/s; MS_C: *M* = 3.62/s, s.d. = 0.69/s; Fig. 2b). Thus, as hypothesized, oxytocin reduced the occurrence of autonomic processing-related microstate C. Furthermore, it decreased the occurrence of visual processing-related microstate B.

Regarding the *coverage* of microstates, we found a significant effect of “treatment” (*F*(1,84) = 6.172, *P* = 0.015, *ETA*^2^ = 0.068) and an interaction effect of “microstate class x treatment” (*F*(3,252) = 2.752, *P* = 0.043, *ETA*^2^ = 0.032). Microstate C revealed, on a marginally significant level, less coverage (*F*(1,84) = 3.810, *P* = 0.054, *ETA*^2^ = 0.043) under oxytocin treatment (*M* = 25.47%, s.d. = 4.01%) compared to placebo treatment (*M* = 27.27%, s.d. = 4.56%). In contrast, microstate D exhibited more coverage (*F*(1,84) = 5.046, *P* = 0.027, *ETA*^2^ = 0.057) under oxytocin treatment (*M* = 24.90%, s.d. = 4.84%) than placebo treatment (*M* = 22.67%, s.d. = 4.35%; Fig. 2c). Thus, as hypothesized, oxytocin decreased the time covered by autonomic processing-related microstate C. Meanwhile, it increased the time covered by attention-related microstate D.

Regarding the *transitions* between microstates, we found a significant interaction effect of “microstate class transition x treatment” (*F*(4.75,398.60) = 2.277, *P* = 0.050, *ETA*^2^ = 0.026). We detected fewer bilateral transitions from microstate B to C under oxytocin-treatment (microstate B → C: placebo: *M* = 0.36%, s.d. = 0.79%; oxytocin: *M* = −0.13%, s.d. = 0.74%; *F*(1,84) = 8.683, *P* = 0.004, *ETA*^2^ = 0.094, Bonferroni-corrected Fig. 2d). In sum, oxytocin decreased transitions from visual processing-related microstate B to autonomic processing-related microstate C.

### Moderation of treatment effects by anxiety-related traits

To corroborate our hypothesis that the observed differences in temporal dynamics of resting networks under oxytocin compared to placebo treatment underlie oxytocin’s anxiolytic effects, we examined whether these differences were more pronounced in participants with high anxiety levels (for details see Supplementary Tab. S2).

**Figure 3:**
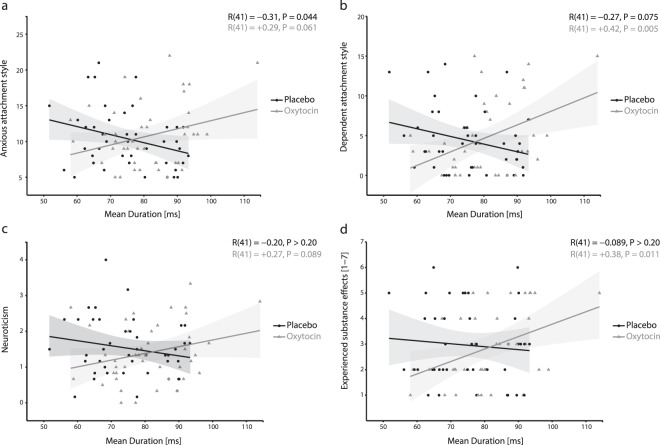
Significant moderations (all *Ps* < 0.046) of oxytocin-induced increases in the temporal stability of resting networks by anxiety-related traits (anxious and dependent attachment styles of the AAS^37,58^ and scale “neuroticism” of the NEO-FFI^36^). Depicted are the correlations (including 95% confidence intervals) between mean microstate duration (ms; x-axis) and anxiety-related traits (**a**: anxious attachment style; **b**: dependent attachment style; **c**: neuroticism) and subjectively experienced substance effects (**d**) separately for participants of the placebo (in black) and oxytocin condition (in grey). Oxytocin-induced increases in the temporal stability of resting networks were more pronounced in participants with higher levels of anxiety-related traits (i.e., more anxious and dependent attachment styles, higher levels of neuroticism) and stronger subjectively experienced substance effects.

Regarding oxytocin’s effect on microstates’ *mean duration*, we identified a moderation by anxious attachment style (*Δ R*^2^ = 8.34%, *F*(1,82) = 7.193, *P* = 0.009), dependent attachment style (*Δ R*^2^ = 10.97%, *F*(1,82) = 8.551, *P* = 0.005), and by neuroticism (*Δ R*^2^ = 4.98%, *F*(1,82) = 4.117, *P* = 0.046). As hypothesized, oxytocin’s effects were more pronounced in people with dependent attachment styles (*R*(41) = 0.42, *P* = 0.005; Fig. 3b), and, on a marginally significant level, in people with anxious attachment styles (*R*(41) = 0.288, *P* = 0.061; Fig. 3a) and high levels of neuroticism (*R*(41) = 0.263, *P* = 0.089; Fig. 3c).

Regarding oxytocin’s effect on the *occurrence of microstate C*, we found a moderation by anxious attachment style (*Δ R*^2^ = 10.45%, *F*(1,82) = 9.072, *P* = 0.004), and by dependent attachment style (*Δ R*^2^ = 10.51%, *F*(1,82) = 6.951, *P* = 0.010). As hypothesized, oxytocin’s effects were more pronounced in people with anxious (*R*(41) = −0.334, *P* = 0.029; Supplementary Fig. S1a) and dependent attachment styles (*R*(41) = −0.397, *P* = 0.008; Supplementary Fig. S1b).

Regarding oxytocin’s effect on the *coverage of microstate C*, we noted a moderation by anxious attachment style (*Δ R*^2^ = 6.44%, *F*(1,82) = 4.846, *P* = 0.031). As hypothesized, oxytocin’s effect was more pronounced in people with an anxious attachment style (*R*(41) = −0.352, *P* = 0.021; Supplementary Fig. S1c). Regarding the *coverage of microstate D*, we found a moderation by dependent attachment style (*Δ R*^2^ = 6.05%, *F*(1,82) = 4.773, *P* = 0.032). Again, this effect was, on a marginally significant level, more pronounced in people with dependent attachment style (*R*(41) = 0.285, *P* = 0.064; Supplementary Fig. S1d).

Regarding oxytocin’s effect on the *transitions from microstate B to microstate C*, we observed a moderation by anxious attachment style (*Δ R*^2^ = 6.26%, *F*(1,82) = 8.585, *P* = 0.004). As hypothesized, this effect was more pronounced in people with an anxious attachment style (*R*(41) = −0.384, *P* = 0.011; Supplementary Fig. S1f).

In sum, we found that several anxiety-related traits moderated oxytocin’s effects, with more pronounced effects in participants possessing high anxiety levels. Together, these findings provide converging evidence that changes in neural resting networks’ temporal dynamics underlie oxytocin’s anxiolytic effects.

### Moderation of treatment effects by subjectively experienced substance effects

Finally, we analyzed whether oxytocin-induced changes in neural networks’ temporal dynamics were moderated by subjectively experienced substance effects (for details see Supplementary Tab. S2). Please note that overall and in line with previous research^38^, there were no differences in subjectively experienced substance effects or in the estimated substance between treatment conditions (both *P* > 0.20).

With regard to microstates’ *mean duration*, we observed a moderation by subjectively experienced substance effects (*Δ R*^2^ = 5.26%, *F*(1,82) = 4.167, *P* = 0.044). As hypothesized, participants revealing stronger oxytocin-induced increases in the temporal stability of resting networks subjectively experienced stronger substance effects (*R*(41) = 0.383, *P* = 0.011; Fig. 3d).

## Discussion

By utilizing a spatio-temporal analysis of 64-channel EEG recorded at rest, the present study provides innovative evidence of oxytocin-induced changes in the temporal dynamics of neural resting networks. In line with oxytocin’s assumed anxiolytic effects, oxytocin administration (1) increased the temporal stability of neural resting networks, (2) decreased the usage and presence of autonomic processing-related microstate C in favor of attention-related microstate D, and (3) decreased communication between the visual-processing related network microstate B and autonomic processing-related microstate C. Anxiety-related traits moderated these changes, with stronger changes in those participants with high rather than low anxiety levels, providing further evidence of oxytocin’s anxiolytic effects. Finally, we observed that the above-mentioned changes correlate with subjectively experienced effects from the oxytocin administration. In sum, the observed oxytocin-induced changes in neural resting networks’ temporal dynamics reveal mechanistic insights into oxytocin’s effects on neuropsychological processes and could potentially inform innovative psychobiological treatment strategies for anxiety-related disorders^39,40^.

Our findings contribute to the scarce knowledge about oxytocin’s effects on neural processing in the temporal domain. Interestingly, we have long known that oxytocin administration affects the processing of very briefly presented stimuli (e.g., 18 milliseconds^41^). As already indicated by this finding, one must rely on neuroscientific methods with high temporal resolution to thoroughly comprehend oxytocin’s neural effects. In doing so, we here show that oxytocin modulates the temporal dynamics of neural resting networks on a milliseconds scale. Our approach differs from previous EEG research reporting complex and context-dependent oxytocin-induced modulations of both event-related potentials^42,43^ and electrophysiological oscillations^44,45^ during the processing of social stimuli. By investigating oxytocin’s effects on the temporal dynamics of neural networks’ intrinsically generated activity while the brain is preparing for forthcoming stimuli processing, this study reveals oxytocin’s general and context-independent effects on neuropsychological processes. To our knowledge, only one other study has investigated oxytocin’s effects on resting EEG. Using a frequency analysis, that study showed that oxytocin decreases cross-frequency coupling across slow and fast oscillations occurring on a seconds scale^22^. In contrast, the spatio-temporal analysis approach we took in this study reveals how oxytocin modulates amplitude-independent and broad-band temporal dynamics of neural resting networks (i.e., their duration, occurrence, coverage, and transitions) occurring on a milliseconds scale^7,25^.

We identified several changes in these neural resting networks’ temporal dynamics. Globally, we found that all networks were present for longer duration under oxytocin vs. placebo treatment, indicating that oxytocin increased the temporal stability of resting networks. This effect might represent an adaptive stabilization of neural processing at rest, as decreased temporal stability of resting networks has been associated with several pathological states such as dementia^46,47^, depression^48^, and schizophrenia^46^. Furthermore, we found that oxytocin decreased the usage and presence of microstate C to benefit increased coverage of microstate D. Previous research has suggested antagonistic functional roles of microstate C and D^33,49^, with microstate C associated with a fronto-insular network involved in interoceptive-autonomic processing^32,33^, and microstate D associated with a fronto-parietal network involved in (re)orienting attention to external stimuli^32,50^. The oxytocin-induced change observed in the balance between microstate C and D toward a relatively increased presence of microstate D might thus indicate a dampening of anxiety-related autonomous processing and a shift from internally- to externally-oriented processing modes at rest^51^. In line with this interpretation, oxytocin decreased the usage of visual processing-related microstate B and its communication to autonomic-processing related microstate C. These findings are also of interest given that oxytocin is being discussed as a potential add-on in the treatment of schizophrenia^52,53^, in which microstate C is more present and microstate D is less present^49^. Together, our findings provide converging evidence of a potential neurobiological mechanism underlying oxytocin’s evolutionarily-conserved role as an anxiolytic facilitator of social interactions^1–3^: while being in an unknown environment at rest, it might increase the temporal stability of neural networks and prepare the brain for the processing of external social instead of internal autonomic information.

Further evidence for these assumed anxiolytic effects comes from the finding that the oxytocin-induced changes we observed in neural networks’ temporal dynamics were more pronounced in participants with high levels of several anxiety-related traits. We found that participants with an anxious attachment style displayed larger increases in networks’ temporal stability, larger decreases in the usage and coverage of the autonomic processing-related microstate C, and larger decreases in the communication from vision-related microstate B to microstate C; participants with a dependent attachment style displayed larger increases in networks’ temporal stability, larger decreases in the usage of microstate C, and larger increases in the coverage of attention-related microstate D. These findings are in line with previous research showing that, in general, individual traits moderate oxytocin’s effects^14^, and, in specific, oxytocin’s effects are stronger in participants with anxious attachment styles^54^. With regard to the potential clinical applications of oxytocin, they suggest that participants with high anxiety levels could benefit more from oxytocin’s anxiolytic effects than those with low anxiety levels. Given that we have detected a positive correlation between increases in networks’ stability and subjectively experienced effects in the oxytocin group, participants with particularly strong neurophysiological changes might even be able to report such beneficial effects.

In sum, our study demonstrates that spatio-temporal analysis of resting EEG provides highly valuable knowledge on oxytocin’s effects on neural networks’ dynamics on a milliseconds scale^55^. Future studies might experimentally modify the environment of resting EEG recordings^56^. For example, one could introduce observation by an experimenter^57^ to investigate whether oxytocin-induced anxiolytic neurophysiological effects are stronger in a social evaluation context. It could also prove worthwhile to extend this research to populations with anxiety-related disorders, in order to study whether oxytocin administration normalizes dysfunctional patterns of resting networks’ temporal dynamics. Future studies might more closely investigate the nature of the correlation between oxytocin-induced changes in neural networks’ temporal dynamics, and subjectively experienced effects, for example by utilizing more specific questions about subjectively experienced anxiolytic effects (e.g., feeling calmer, less tense). If future evidence solidifies that changes in neural networks’ temporal dynamics underlie oxytocin’s anxiolytic effects, they could inform clinical applications of oxytocin to treat anxiety-related disorders.

## Methods

### Participants

All participants were free of a current or previous history of neurological or psychiatric disorders, alcohol or drug abuse, or allergies to the preservatives used in nasal sprays. Ninety-one healthy male participants took part in this experiment. Five participants had to be excluded from further analysis because of excessive artifacts in their EEG recordings (<50% of data were available after automatic and manual artifact corrections), leaving a sample of 86 participants for analysis. Mean age was 23.7 y (s.d. = 4.59 y, range: 18–39 y). The Ethics Committee of the University of Freiburg approved this study, which was conducted according to the principles expressed in the Declaration of Helsinki. All procedures were carried out with the adequate understanding and informed consent of the participants.

### Procedure

There were two appointments: during the first appointment, participants completed anxiety-related questionnaires (scale “neuroticism” of the NEO-FFI^36^; anxious and dependent attachment styles of the AAS^37,58^). The second appointment took place in the EEG laboratory. In a randomized, placebo-controlled, double-blind between-group design, participants received either 24 international units (IU) of oxytocin (N = 43) or placebo (N = 43) via intranasal administration^59–61^, following a well-established protocol^27,31^. Participants were then guided to an electrically shielded cabin in which 64-channel EEG measurements were prepared. The resting EEG protocol consisted of 20-s eyes open followed by 40-s eyes closed, repeated five times. This resting state paradigm has been routinely used in resting EEG research to minimize fluctuations in participants’ vigilance state^62–64^, because participants can become drowsy already after 3 minutes of recording resting state brain activity if there is no alternation of eyes-open/eyes-closed periods^65^. The instructions about eye opening/closing were given via intercom. To exclude the possibility that instruction delivery confounds the resting state during the eyes-closed periods, we delivered instructions at the beginning and end of the eyes-open periods. Forty-five minutes after intranasal administration^27,30,31^, we measured each participant’s electroencephalogram during the resting state. We also collected subjective ratings on their experience of substance effects (7-point Likert Scale ranging from “1 = no effect at all” to “7 = very strong effect”). After these ratings, participants performed a social decision-making paradigm which will be analyzed elsewhere.

### EEG recording

The EEG was recorded with a 64-channel recording system (Brainamp with actiCAP, Brain Products Gmbh, Munich) according to the 10–10 system montage^66^. Scalp impedance was kept below 10 kΩ. FCz served as the reference electrode, AFz as the ground electrode. Horizontal and vertical electrooculographic signals were recorded with two additional electrodes at the left and right outer canthi and one electrode at the left infraorbital. The EEG was online band-pass filtered between 0.1 and 100 Hz, and the data digitized with a sampling rate of 500 Hz.

### EEG Preprocessing

EEG data were preprocessed using Brain Vision Analyzer (Version 2.0.1.327; Brain Products GmbH, Munich). Only the 200 s eyes-closed periods were used for the analysis, because the influence of external visual stimulus processing and confounding eye blinks is minimized^67,68^. Data were band-pass filtered (high-pass 2 Hz, low-pass 20 Hz^25^) and re-derived to average reference. Ocular correction was conducted via a semi-automatic Independent Component Analysis (ICA)-based correction process. EEG signals with excessive noise were replaced by using a linear interpolation of adjacent electrodes. After an automatic artifact rejection (maximum amplitude: ±100 *μV*), data were visually examined by two independent raters to eliminate residual artifacts. Finally, artifact-free data were segmented into 2-s epochs for further analyses (*M* = 171 s, s.d. = 18 s, range: 86–192 s).

### EEG microstate analysis

First, following the standard procedure^6,69,70^, the maps at the momentary peaks of the Global Field Power (i.e., maximum voltage values at all electrodes at that time point that represent time points of optimal signal-to-noise ratio) were extracted and submitted to a modified spatial cluster analysis using the atomize-agglomerate hierarchical clustering (AAHC^71,72^) method. In line with previous research^72^ we identified the four most dominant cluster maps in every single subject that represent the four archetypal EEG resting networks. In the second step, the cluster maps identified in each subject were submitted for a second cluster analysis to identify the dominant maps across all participants in each treatment group, yielding separate grand mean maps for participants under placebo and oxytocin treatment. Third, this analysis was repeated for the whole sample, yielding grand mean maps for the whole sample which were then sorted according to the standard labeling^25^ (microstate A exhibits a left-right orientation, microstate B exhibits a right-left orientation, microstate C exhibits an anterior-posterior orientation, and microstate D exhibits a fronto-central maximum). Fourth, the grand mean maps of the placebo and oxytocin conditions were sorted according to the grand mean maps of the entire sample. Fifth, the maps of each individual were sorted according to the grand mean maps of the specific treatment group on the basis of spatial correlations. Sixth, and finally, the GFP peaks of individual EEG data were assigned to the individually identified cluster maps to which they fitted best. This assignment was linearly interpolated to the time periods between the GFP peaks, yielding a continuous temporal stream of microstates occurring in each individual. We extracted four main microstates characteristics of interests from this last step: (1) The average microstate duration, representing an index of the temporal stability of the underlying resting network, (2) the average frequency of occurrence for each microstate independent of its individual duration, representing an index of the relative usage of the underlying resting network, (3) the total percentage of time during which a given microstate is dominant, representing the total presence of the underlying neural network, and (4) the number of observed transitions normalized for the overall count of transitions (“more transitions than expected from the number of occurrence of each microstate”) from one microstate to any other microstate which reveal sequential activation of and communication between the underlying neural networks.

### Statistical analysis

To guarantee that the same EEG resting networks were present in both the oxytocin and placebo group, we first compared the topography of the mean maps of the two treatment groups using a randomization test (TANOVA), as implemented in Ragu software^73–75^. Next, we compared microstate characteristics (duration, occurrence, coverage, transitions) between treatment groups calculating ANOVAs with the between-subjects factor “treatment” (oxytocin vs. placebo) and within-subjects factor “microstate class” (microstates A-D) or “microstate class transition” (twelve transitions, e.g., microstate A→B, microstate A→C, microstate A→D). We used parametric ANOVAs and report two-sided p-values. If the variances of the differences between treatment levels were unequal (Mauchly’s test for sphericity:  P > 0.05) we report Greenhouse-Geisser corrected P-values. In the main manuscript, we report only significant effects involving the factor “treatment” (for significant effects involving the factor “microstate class”, please see Supplementary Results). In case of significant main or interaction effects, we calculated one-way ANOVAs comparing microstates characteristics between treatment groups; as we had no specific a priori hypotheses for microstates transitions, we applied Bonferroni-correction to these findings. For all significant effects, we also tested for significant moderation effects by anxiety-related personality traits and subjectively experienced substance effects using PROCESS^76^.

## Supplementary information

Supplementary Material

## Data Availability

All data generated during and/or analyzed during the current study are available from the corresponding authors on reasonable request.
