# Supplementary material for: Oxytocin modulates the temporal dynamics of resting EEG networks
Source: Sci Rep. 2019 Sep 26;9:13418. doi: 10.1038/s41598-019-49636-6 (PMC6763457; doi:10.1038/s41598-019-49636-6)
Supplement: Supplementary file 1 — Supplementary Material [file 41598_2019_49636_MOESM1_ESM.docx]

Supplementary Information

**Oxytocin modulates the temporal dynamics of resting EEG networks**

Bastian Schiller*^1, 2^, Thomas Koenig^3^, Markus Heinrichs^1,2^

^1^Department of Psychology, Laboratory for Biological and Personality Psychology, University of Freiburg, DE-79104, Freiburg, Germany

^2^Freiburg Brain Imaging Center, University Medical Center, University of Freiburg, DE-79104, Freiburg, Germany

^3^Translational Research Center, University Hospital of Psychiatry, University of Bern, CH-3000, Bern, Switzerland

*Corresponding author: [schiller@psychologie.uni-freiburg.de](mailto:schiller@psychologie.uni-freiburg.de)

**Supplementary Results**

To compare the temporal dynamics of resting networks (*duration, occurrence, coverage, transitions*) between treatment groups, we calculated ANOVAs with the between-participants factor “treatment” (oxytocin vs. placebo) and the within-participants factor “microstate class” (four levels: microstate A-D) or “microstate class transition” (twelve levels: from microstate A-D [4] to microstate A-D [3]). We here report main effects of “microstate class” which we did not report in the main manuscript. Regarding the *duration* of microstates, we observed a significant effects of “microstate class” (*F*(3,252)= 3.35, *P* = 0.020, *ETA^2^* = 0.038). Post-hoc LSD-tests revealed a shorter duration of microstate D (*M* = 75.22ms, s.d. = 14.28ms) compared to microstates A (*M* = 78.78ms, s.d. = 13.00ms; *P* = 0.004) and C (*M* = 78.60ms, s.d. = 13.61ms; *P* = 0.005). Regarding the *occurrence* of microstates, we observed a significant effects of “microstate class” (*F*(3,252) = 4.90, *P* = 0.003, *ETA^2^* = 0.055). Post-hoc LSD-tests revealed that microstate C (*M* = 3.42/s, s.d. = 0.67/s) occurred more frequently compared to microstates A (*M* = 3.27/s, s.d. = 0.65/s; *P* = 0.027), B (*M* = 3.20/s, s.d. = 0.64/s; *P* = 0.001), and D (*M* = 3.21/s, s.d. = 0.58/s; *P* = 0.001). Regarding the *coverage* of microstates, we observed a significant effects of “microstate class” (*F*(3,252) = 3.35, *P* = 0.016, *ETA^2^* = 0.040). Post-hoc LSD-tests revealed that microstate C (*M* = 26.37%, s.d. = 0.043%) had a higher coverage compared to microstate B (*M* = 24.45%, s.d. = 0.054%; *P* = 0.030) and D (*M* = 23.79%, s.d. = 0.047%; *P* < 0.001). Regarding the *transitions* of microstates, we observed a significant effects of “microstate class” (F(4.75,398.60) = 2.95, P = 0.014, *ETA^2^* = 0.034). Post-hoc LSD-tests revealed that the transition microstate A🡪B (M = -0.35%, s.d. = 0.90%) occurred less often than the transitions microstate A🡪C (*M* = 0.22%, s.d. = 0.79%; *P* = 0.001), A🡪D (*M* = 0.12%, s.d. = 0.82%; *P* = 0.005), B🡪A (*M* = -0.02%, s.d. = 0.90%; *P* = 0.001), B🡪C (*M* = 0.12%, s.d. = 0.80%; *P* = 0.001), C🡪A (*M* = 0.03%, s.d. = 0.97%; *P* = 0.019), C🡪B (*M* = 0.19%, s.d. = 0.96%; *P* < 0.001), D🡪A (*M* = -0.04%, s.d. = 0.82%; *P* = 0.034), D🡪B (*M* = -0.08%, s.d. = 0.77%; *P* = 0.05), and D🡪C (*M*=0.11%, s.d. = 0.90%; *P* < 0.001); the transition microstate A🡪C (*M* = 0.22%, s.d. = 0.79%) occurred more often than the transition microstate B🡪D (*M* = -0.11%, s.d. = 0.95%; *P* = 0.001), C🡪D (M = -0.23%, s.d. = 1.03%; *P* = 0.004), and D🡪B (*M* = -0.08%, s.d. = 0.77%; *P* = 0.003); the transition microstate A🡪D (*M* = 0.12%, s.d. = 0.82%) occurred more often than the transition microstate C🡪D (*M* = -0.23%, s.d. = 1.03%; *P* = 0.027); the transition microstate B🡪A (*M* = -0.02%, s.d. = 0.90%) occurred more often than the transition microstate C🡪D (*M* = -0.23%, s.d. = 1.03%; *P* = 0.045); the transition microstate B🡪C (*M* = 0.12%, s.d. = 0.80%) occurred more often than the transition microstate C🡪D (*M* = -0.23%, s.d. = 1.03%; *P* = 0.029); the transition microstate B🡪D (*M* = -0.11%, s.d. = 0.95%) occurred less often than the transition microstate C🡪B (*M* = 0.19%, s.d. = 0.96%; *P* = 0.05); the transition microstate C🡪B (*M* = 0.19%, s.d. = 0.96%) occurred more often than the transitions microstate C🡪D (*M* = -0.23%, s.d. = 1.03%; *P* = 0.027), D🡪A (*M* = -0.04%, s.d. = 0.82%; *P* = 0.035), and D🡪B (*M* = -0.08%, s.d. = 0.77%; *P* = 0.039); and the transition microstate C🡪D (*M* = -0.23%, s.d. = 1.03%) occurred less often than the transition microstate D🡪C (*M* = 0.11%, s.d. = 0.90%; *P* < 0.001).

**Supplementary Table S1 Descriptive Statistics of microstates’ characteristics**

|  |  | Duration (ms) | | Occurrence (Times/s) | | Coverage (%) | |
| --- | --- | --- | --- | --- | --- | --- | --- |
| Sample | Microstate | *M* | s.d. | *M* | s.d. | *M* | s.d. |
| All (n=86) | A | 78.78 | 13.00 | 3.27 | 0.65 | 25.40 | 5.02 |
|  | B | 77.52 | 13.72 | 3.20 | 0.64 | 24.45 | 5.38 |
|  | C | 78.60 | 13.61 | 3.42 | 0.67 | 26.37 | 4.36 |
|  | D | 75.22 | 14.28 | 3.21 | 0.58 | 23.79 | 4.71 |
|  | A-D | 78.16 | 11.72 | 13.09 | 2.03 | 25.00 |  |
| Placebo (n=43) | A | 75.30 | 12.53 | 3.34 | 0.70 | 24.82 | 5.35 |
|  | B | 75.00 | 12.91 | 3.40 | 0.66 | 25.23 | 5.51 |
|  | C | 76.71 | 13.90 | 3.63 | 0.69 | 27.27 | 4.56 |
|  | D | 71.05 | 12.90 | 3.24 | 0.62 | 22.67 | 4.56 |
|  | A-D | 75.20 | 11.37 | 13.61 | 2.13 | 25.00 |  |
| Oxytocin (n=43) | A | 82.26 | 12.65 | 3.20 | 0.59 | 25.97 | 4.65 |
|  | B | 80.03 | 14.19 | 2.99 | 0.56 | 23.68 | 5.20 |
|  | C | 80.50 | 13.21 | 3.21 | 0.58 | 25.47 | 4.01 |
|  | D | 79.40 | 14.50 | 3.17 | 0.53 | 24.90 | 4.84 |
|  | A-D | 81.11 | 11.45 | 12.57 | 1.80 | 25.00 |  |

**Supplementary Table S2 Moderation of oxytocin-induced changes of microstates characteristics**

| MS | Characteristic | AAS_A | AAS_D | AAS_C | NEO_N | NEO_E | NEO_O | NEO_V | NEO_G | ESE |
| --- | --- | --- | --- | --- | --- | --- | --- | --- | --- | --- |
| A-D | Duration | **.009** | .005 | **.162** | .046 | .133 | >.20 | >.20 | **>.20** | **.044** |
| B | Occurrence | >.20 | .141 | >.20 | >.20 | .127 | >.20 | >.20 | .133 | .051 |
| C | Occurrence | **.004** | .010 | .125 | .062 | >.20 | >.20 | >.20 | >.20 | >.20 |
| C | Coverage | .031 | >.20 | >.20 | >.20 | .073 | >.20 | >.20 | >.20 | >.20 |
| D | Coverage | **>.20** | .032 | >.20 | .071 | .041 | **>.20** | .036 | .067 | >.20 |
| B -> C | Transition | .004 | >.20 | >.20 | >.20 | .077 | >.20 | .136 | >.20 | >.20 |
| C -> B | Transition | >.20 | >.20 | >.20 | >.20 | >.20 | >.20 | >.20 | >.20 | >.20 |
| B -> D | Transition | .119 | >.20 | >.20 | >.20 | >.20 | >.20 | >.20 | >.20 | >.20 |

Shown are the *P*-values of interaction effects testing for a moderation of oxytocin-induced changes of microstates characteristics by anxiety-related traits and subjectively experienced substance effects using PROCESS^64^. AAS_A: Anxious attachment style from the Adult Attachment Scale^31,33^; AAS_D: Dependent attachment style from the Adult Attachment Scale^31,33^; AAS_C: Close attachment style from the Adult Attachment Scale^31,33^; NEO_N: scale “Neuroticism” of the NEO-FFI^30^; NEO_E: scale “Extraversion” of the NEO-FFI^30^; NEO_O: scale “Openness to experience” of the NEO-FFI^30^; NEO_A: scale “Agreeableness” of the NEO-FFI^30^; NEO_C: scale “Conscientiousness” of the NEO-FFI^30^. ESE: Subjectively experienced substance effects (7-point Likert Scale ranging from “1 = no effect at all” to “7 = very strong effect”).

­­
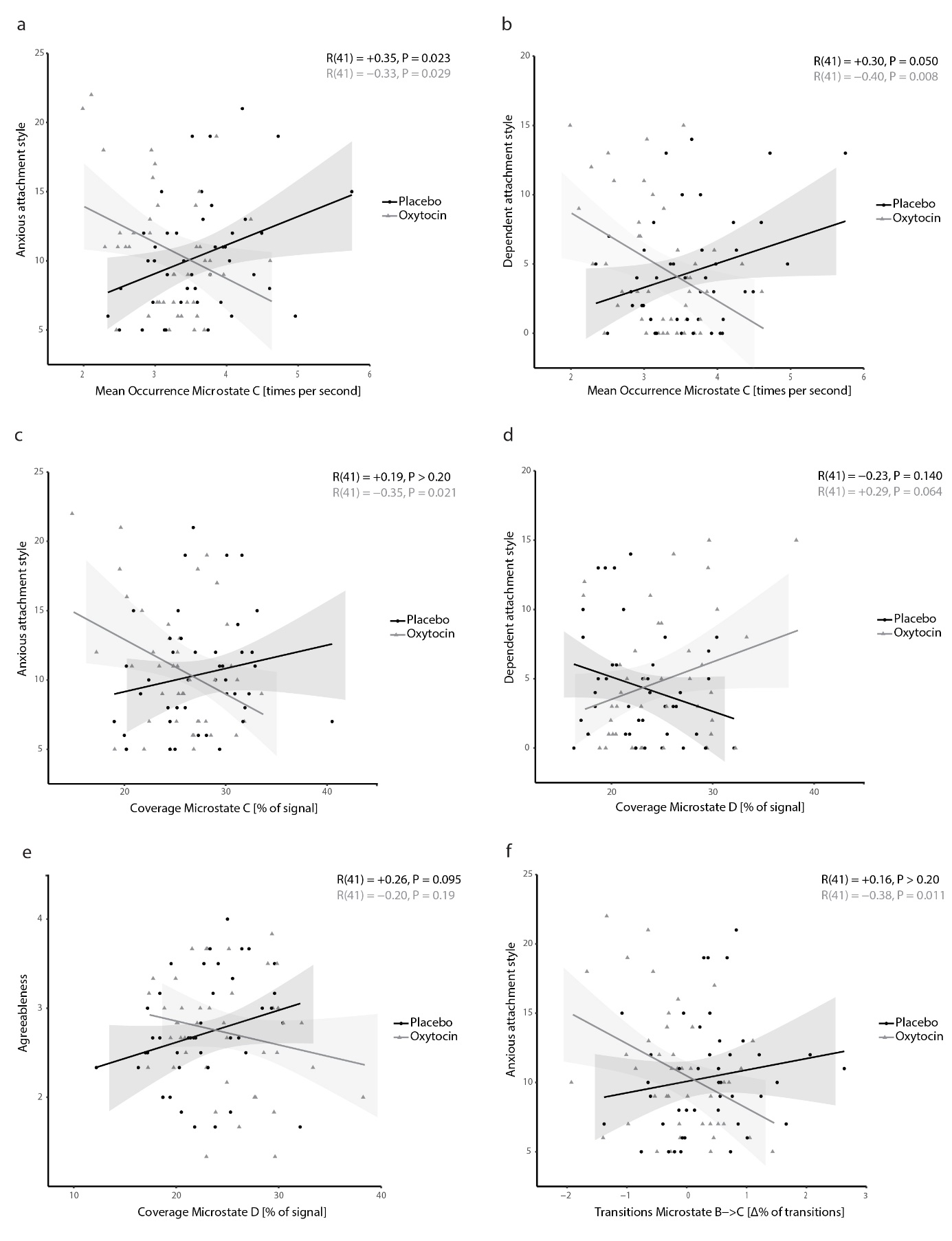


**Figure S1**: Moderation of oxytocin-induced changes in the temporal characteristics of resting networks (anxious and dependent attachment styles of the AAS^33^ and scale “neuroticism” of the NEO-FFI^30^). Depicted are the correlations (including 95% confidence intervals) between microstate characteristics (**a&b**: mean occurrence microstate C; **c**: coverage microstate C; **d&e**: coverage microstate D; **f**: transitions microstate B->C) and anxious attachment style (**a, c, f**), dependent attachment style (**b, d**), and agreeableness (**e**), separately for participants of the placebo (in black) and oxytocin condition (in grey). Oxytocin-induced increases in the temporal stability of resting networks were more pronounced in participants with higher levels of anxiety-related traits (i.e., more anxious and dependent attachment styles, higher levels of neuroticism). We also found that the oxytocin-induced increase in coverage of microstate D was more pronounced in participants with lower levels of agreeableness.
